# Supplementary material for: Abdominal Symptoms and Colonic Diverticula in Marfan’s Syndrome: A Clinical and Ultrasonographic Case Control Study
Source: J Clin Med. 2020 Sep 28;9(10):3141. doi: 10.3390/jcm9103141 (PMC7599469; doi:10.3390/jcm9103141)
Supplement: Supplementary file 1 [file jcm-09-03141-s001.pdf]

Appendix 1

Questionnaire

The following questionnaire aims to investigate the presence of non-specific gastrointestinal symptoms and and symptoms suggesting diverticulosis colonic. The results of the questionnaire will then be used for scientific studies, in an absolutely anonymous and confidential form.

Patient n._______

Name_________________________ Surname_______________________

A) Age:________ Date of Birth:__________________

B) Gender: [M] [F]

C) High (cm):_________ Weight (kg):__________

D) Have you ever performed a colonoscopy / barium enema or CT colonography?

[No]

[Yes]: Why? ____

Results? ____

E) Have you ever performed a fecal occult blood test?

[No] [Yes]: Results? ____

F) Have you ever performed an abdominal ultrasound?

[No] [Yes]: Results? ____

G) Have you ever been submitted to an abdominal surgery ?

[No] [Yes]: why? ____

H) Please report which of the following symptoms you complained for more than 7 days (even not consecutive) in the last year requiring, visit or any treatment (including OTC)

1. **Abdominal pain** **[Yes]** **[No]**
2. **Bloating** **[Yes]** **[No]**
3. **Diarrhea** **[Yes]** **[No]**
4. **Constipation** **[Yes]** **[No]**
